# Дифференцированная карцинома щитовидной железы у детей и подростков

**DOI:** 10.14341/probl13669

**Published:** 2026-01-18

**Authors:** Е. В. Нагаева, Э. Б. Бричева, Д. Н. Бровин, А. В. Аникиев, А. М. Артемова, Ф. М. Абдулхабирова, А. Ю. Абросимов, Д. А. Пастухова, Л. С. Урусова, К. Ю. Слащук, М. С. Шеремета, И. Р. Минниахметов, О. Б. Безлепкина, А. В. Петеркова

**Affiliations:** Национальный медицинский исследовательский центр эндокринологииРоссия; Endocrinology Research CentreRussian Federation

**Keywords:** узловой зоб, дифференцированная карцинома щитовидной железы, дети, хирургическое лечение, рецидив злокачественного новообразования щитовидной железы, nodular goiter, differentiated thyroid carcinoma, children, surgical treatment, recurrence of malignant thyroid neoplasm

## Abstract

**ОБОСНОВАНИЕ:**

ОБОСНОВАНИЕ. Узловые образования щитовидной железы (ЩЖ) у детей встречаются относительно редко, однако риск их злокачественного характера значительно выше, чем у взрослых. Оптимальный объем хирургического вмешательства, показания к радиойодтерапии (РЙТ) и роль молекулярно-генетического тестирования у детей с карциномой ЩЖ остаются предметом дискуссии.

**ЦЕЛЬ:**

ЦЕЛЬ. Изучить особенности течения дифференцированной карциномы щитовидной железы (ДКЩЖ) у детей, а также результаты хирургического и комбинированного лечения по данным 10-летней клинической практики ГНЦ РФ ФГБУ «НМИЦ эндокринологии им. академика И.И. Дедова» Минздрава РФ (далее — ЭНЦ).

**МАТЕРИАЛЫ И МЕТОДЫ:**

МАТЕРИАЛЫ И МЕТОДЫ. В ретроспективное одноцентровое исследование включены 980 пациентов детского и подросткового возраста, оперированных по поводу узловых образований в ЩЖ в 2015–2024 гг. Протокол обследования включал сбор жалоб и анамнеза, физикальное обследование, ультразвуковое исследование ЩЖ, тонкоигольную аспирационную биопсию, патоморфологический анализ послеоперационного материала. Детям с отягощенной наследственностью и/или подозрением на наличие синдромов, ассоциированных с возникновением карциномы ЩЖ, было выполнено молекулярно-генетическое исследование (МГИ). Медиана длительности динамического наблюдения для детей с ДКЩЖ составила 12 месяцев [1,0; 36,0].

**РЕЗУЛЬТАТЫ:**

РЕЗУЛЬТАТЫ. Злокачественные новообразования ЩЖ выявлены у 506 пациентов, из них у большинства — дифференцированная карцинома щитовидной железы (n=472). Наиболее распространенным гистологическим типом была папиллярная карцинома (n=448; 88,5%). Метастатическое поражение регионарных лимфатических узлов (ЛУ) имелось у 21% детей. Адъювантная РЙТ проведена 48,5% пациентам, при этом отдаленные метастазы в легкие выявлены в 5,7% случаев. Рецидивы ДКЩЖ зарегистрированы у 5,1% детей; у 16,5% заболевание сохранялось после первичного лечения в виде биохимической и/или структурной персистенции. МГИ проведено 66 детям, патогенные варианты выявлены у 53,1%, наиболее часто — в генах DICER1, PTEN и APC.

**ЗАКЛЮЧЕНИЕ:**

ЗАКЛЮЧЕНИЕ. Дифференцированная карцинома щитовидной железы у детей характеризуется рядом клинических и молекулярно-генетических особенностей, что определяет необходимость специализированного мультидисциплинарного подхода к их ведению. Высокий риск злокачественности в узлах, частая регионарная диссеминация и особенности молекулярного профиля аргументируют необходимость ранней диагностики, интеграции молекулярного тестирования и персонализированного выбора объема хирургического вмешательства в условиях профильных центров.

## Abstract (en)

**BACKGROUND:**

BACKGROUND: Thyroid nodules in children are relatively rare; the risk of malignancy is considerably higher compared to adults. The optimal extent of surgery, the indications for radioactive iodine therapy (RAI), and the role of molecular genetic testing in children with thyroid carcinoma remain a matter of debate.

**AIM:**

AIM: To investigate the clinical course of differentiated thyroid carcinoma (DTC) in children, as well as the outcomes of surgical and combined treatment based on ten years clinical experience at the Endocrinology Research Centre.

**MATERIALS AND METHODS:**

MATERIALS AND METHODS: This retrospective single-center study included 980 pediatric and adolescent patients who underwent surgery for thyroid nodules between 2015 and 2024. The diagnostic protocol comprised history taking, physical examination, thyroid ultrasound, fine-needle aspiration biopsy, and postoperative histopathological evaluation. Molecular genetic testing was performed in children with a family history or suspected syndromic forms of thyroid carcinoma. The median follow-up duration for patients with DTC was 12 months [1.0; 36.0].

**RESULTS:**

RESULTS: Malignant thyroid tumors were identified in 506 patients (51.6%), the majority of them — differentiated thyroid carcinoma (DTC) (n=472). The most common histological type was papillary thyroid carcinoma (n=448; 88,5%). Metastatic involvement of regional lymph nodes was present in 21% of children. Adjuvant RAI was performed in 48.5% of patients; distant metastases to the lungs were detected in 5.7% of cases. Recurrences were registered in 5.1% of children; in 16.5% of children the disease persisted after primary treatment in the form of biochemical and/or structural persistence. MGI was performed in 66 children; pathogenic variants were detected in 53.1%, most frequently in the DICER1, PTEN, and APC genes.

**CONCLUSION:**

CONCLUSION: Differentiated thyroid carcinoma in children is characterized by a number of clinical and molecular genetic features, which determines the need for a specialized multidisciplinary approach to their management. The high risk of malignancy in nodules, frequent regional dissemination and peculiarities of the molecular profile argue for the necessity of early diagnosis, integration of molecular testing and personalized choice of the volume of surgical intervention in the conditions of specialized centers.

## ОБОСНОВАНИЕ

Узловые образования щитовидной железы (ЩЖ) у детей представляют клинико-морфологически гетерогенную группу заболеваний как доброкачественного, так и злокачественного характера. Несмотря на относительно низкую распространенность в детской популяции (0,2–5,1%), риск злокачественности в узлах у детей достигает 22–26%, что в 2,5 раза превышает аналогичный показатель у взрослых [1–3]. В педиатрии наиболее частой злокачественной опухолью является дифференцированная карцинома щитовидной железы (ДКЩЖ), хотя в структуре детской онкологии она остается редкой патологией [4][5].

По данным международных регистров, за последние два десятилетия заболеваемость ДКЩЖ у детей демонстрирует устойчивый ежегодный рост на 3,6–4,4%, главным образом за счет увеличения числа случаев папиллярной карциномы в возрастной группе старше 10 лет [6][7]. Аналогичные тенденции характерны и для Российской Федерации: среди детей до 14 лет включительно заболеваемость составляет 0,05–0,12 на 100 000 детского населения, тогда как в 15–17 лет достигает 0,44–1,1, причем подавляющее большинство случаев регистрируется у девочек подросткового возраста (соотношение девочки:мальчики 2:1–6:1) [5][8][9]. Рост выявляемости во многом обусловлен внедрением высокочувствительных методов визуализации, прежде всего ультразвукового исследования высокой разрешающей способности, увеличением распространенности факторов риска, включая перенесенное облучение головы и шеи, дефицит йода, аутоиммунные тиреоидиты, а также улучшением диагностики наследственных синдромов, ассоциированных с возникновением карциномы ЩЖ [10–13].

Дифференцированная карцинома щитовидной железы у детей по сравнению со взрослыми имеет ряд особенностей: опухоль часто выявляется на более поздних стадиях, нередко сопровождается двусторонним мультифокальным ростом, массивным поражением регионарных лимфоузлов и отдаленными метастазами в легкие, которые регистрируются у 30–35% пациентов уже при постановке диагноза [1][8][14]. У детей младше 10 лет инкапсулированные новообразования встречаются лишь в 13% случаев, тогда как в возрасте 11–15 лет — в 21%, что отражает более агрессивный и инвазивный характер роста узловых образований ЩЖ у пациентов младшего возраста [14]. Метастазы в легкие, по данным некоторых исследований, сохраняют функциональную активность и высокую чувствительность к терапии радиоактивным йодом [1][15].

Отдельного внимания заслуживают патоморфологические особенности детских опухолей, которые, в отличие от взрослых, чаще имеют солидно-фолликулярный или классический подтип папиллярной карциномы [14][16].

ДКЩЖ у детей является новообразованием с характерными клинико-морфологическими и молекулярно-генетическими особенностями, что определяет необходимость специализированного подхода к ее диагностике и лечению. Вклад в изучение данной патологии вносят федеральные центры, аккумулирующие значительное количество пациентов [17]. С октября 2015 г. в ГНЦ РФ ФГБУ «НМИЦ эндокринологии им. академика И.И. Дедова» Минздрава России функционирует детское хирургическое отделение [18], в котором за последние три года стабильно выполняется более 200 операций на щитовидной железе ежегодно (рис. 1). По данным формы №14 федерального статистического наблюдения «Сведения о деятельности подразделений медицинской организации, оказывающих медицинскую помощь в стационарных условиях», за последние шесть лет в Российской Федерации проведено 3119 хирургических вмешательств на щитовидной железе у детей, из них треть операций (n=1053) выполнена в ЭНЦ (рис. 2).

**Figure:**
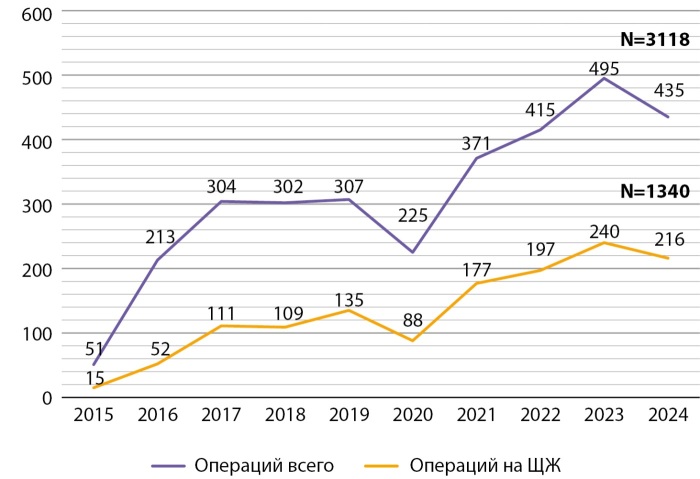
Рисунок 1. Динамика всей операционной активности и операций на щитовидной железе в детском хирургическом отделении ГНЦ ФГБУ «НМИЦ эндокринологии им. академика И.И. Дедова» МЗ РФ за 2015–2024 гг.

**Figure:**
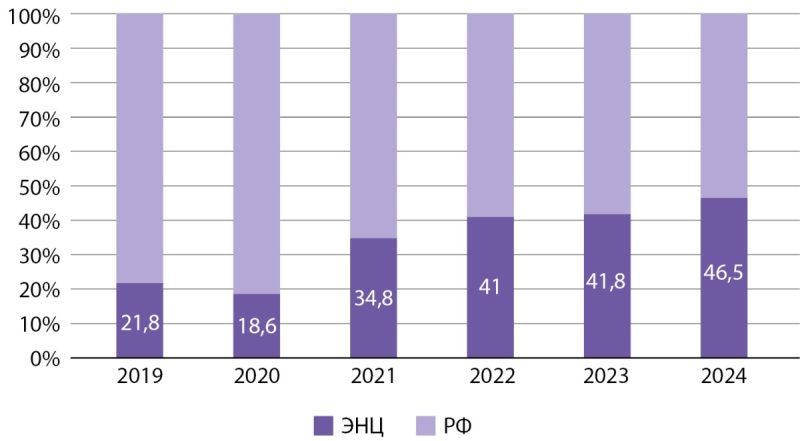
Рисунок 2. Процент проведенных оперативных вмешательств на щитовидной железе у детей в ЭНЦ среди всех оперативных вмешательств на щитовидной железе у детей в РФ по данным формы №14 федерального статистического наблюдения «Сведения о деятельности подразделений медицинской организации, оказывающих медицинскую помощь в стационарных условиях» за последние 6 лет.

## ЦЕЛЬ ИССЛЕДОВАНИЯ

Изучить особенности течения дифференцированной карциномы щитовидной железы у детей, а также результаты хирургического и комбинированного лечения по данным десятилетней клинической практики в ЭНЦ.

## МАТЕРИАЛЫ И МЕТОДЫ

## Место и время проведения исследования

Место проведения. ГНЦ РФ ФГБУ «НМИЦ эндокринологии им. академика И.И. Дедова» Минздрава России.

Время исследования. 2015–2024 гг.

## Изучаемые популяции

Пациенты детского и подросткового возраста, прооперированные в детском хирургическом отделении ЭНЦ по поводу узловых образований ЩЖ.

Критерии включения:

Критерии исключения:

## Способ формирования выборки

Сплошной — в исследование включены все пациенты, соответствующие критериям включения и не соответствующие критериям исключения, за указанный период.

## Дизайн исследования

Одноцентровое, наблюдательное, одномоментное, ретроспективное.

## Методы

Протокол обследования. Комплексное обследование включало: оценку жалоб и анамнеза, физикальное обследование, ультразвуковое исследование щитовидной железы и регионарных лимфатических узлов, тонкоигольную аспирационную биопсию (ТАБ) узловых образований, а также вторично измененных лимфатических узлов шеи со смывом на тиреоглобулин, цитологическое исследование аспирированного материала с оценкой по системе классификации цитопатологии щитовидной железы Bethesda, патоморфологическое исследование послеоперационного материала, молекулярно-генетическое тестирование методом секвенирования нового поколения (next generation sequencing, NGS), анализ объема и тактики хирургического лечения, оценку показаний и проведение радиойодтерапии.

Ультразвуковое исследование (УЗИ) щитовидной железы проводилось на ультразвуковом сканере (Voluson E8, GE; Тoshiba Aplio 500) с использованием линейных датчиков с частотой 11 МГц и 7–18 МГц. Сканирование ЩЖ осуществлялось в В-режиме и с применением цветового допплеровского картирования. Производилось измерение трех размеров обеих долей ЩЖ (длина, ширина и передне-задний размер), объем ЩЖ вычислялся по формуле J. Brunn (1981 г.) [ширина правой доли (см) х длина правой доли (см) х толщина правой доли (см) + ширина левой доли (см) х длина левой доли (см) х толщина левой доли (см)] х 0,479. Оценивалась структура ЩЖ, эхогенность, васкуляризация, наличие узловых образований, их структура. УЗ-признаки узлов классифицировались по системе EU-TIRADS (2017 г.).

Тонкоигольная аспирационная биопсия узловых образований ЩЖ проводилась методикой «free-hand» под контролем УЗИ иглой 22G. Аспирированный материал помещали на покровное стекло, высушивали. Цитологические стекла были окрашены по Романовскому-Гимзе (НПФ Абрис+, Россия).

Цитологическое исследование. Просмотр цитологических стеклопрепаратов осуществлялся на микроскопах Leica DM LS2 и ZEISS Primo Star с увеличением ×4, ×10, ×20, ×40.

Молекулярно-генетическое исследование проводилось у детей с отягощенной наследственностью и/или подозрением на наличие синдромов, ассоциированных с возникновением карциномы ЩЖ. Исследование выполнялось методом массового параллельного секвенирования (NGS) на платформе Illumina (2×100 п.о.) с использованием библиотечных наборов KAPA HyperPlus и KAPA HyperCapture (Roche). Анализ включал выравнивание на референсный геном человека (HG38), фильтрацию, аннотацию вариантов и оценку их патогенности с использованием SIFT, PolyPhen-2, PROVEAN, CADD, SpliceAI, AdaBoost. Патогенность оценивалась в соответствии с международными и российскими клиническим рекомендациям [19][20].

Хирургическая тактика. Объем хирургического лечения определялся на основании цитологического заключения по системе Bethesda, клинико-инструментальных характеристик узловых образований, а также молекулярно-генетических данных.

При наличии у пациента герминальных мутаций, ассоциированных с высоким риском развития ДКЩЖ или прогрессирующим ростом узловых образований в ЩЖ (DICER1, PTEN, APC и др.), радикальная операция (тиреоидэктомия с центральной лимфаденэктомией) рассматривалась как предпочтительный вариант для профилактики повторных вмешательств и снижения количества операций по поводу узлов ЩЖ.

Патоморфологическое исследование послеоперационного материала проводилось согласно общепринятой стандартной методике: образцы ткани фиксировали в забуференном растворе формалина, затем дегидратировали в батарее спиртов восходящей концентрации и заливали в парафин. Срезы щитовидной железы толщиной ≈3,5 μm помещали на гистологические предметные стекла с последующей депарафинизацией и окрашиванием образцов гематоксилином и эозином в гистостейнере ST5010 AXL («Leica», Великобритания).

Стратификация риска рецидива/прогрессирования карциномы щитовидной железы. Все пациенты с карциномой щитовидной железы были оценены в соответствии со стратификацией риска рецидива/прогрессирования, предложенной Американской тиреоидологической ассоциацией (ATA, 2015), что позволило унифицировать подход к интерпретации клинических исходов и сопоставить наши результаты с современными международными данными [21].

Показания к проведению радиойодтерапии. Адъювантная радиойодтерапия была рекомендована детям из группы промежуточного и высокого риска рецидива/прогрессирования карциномы щитовидной железы согласно критериям ATA [21]. Повторные курсы РЙТ назначались при выявлении сохраняющейся или рецидивной йоднакапливающей опухолевой ткани, а также при метастатическом процессе без достижения полной ремиссии. Решение об их проведении принималось индивидуально на основании динамики тиреоглобулина у пациентов после тиреоидэктомии, данных УЗИ/КТ и сцинтиграфии.

Статистический анализ. Статистическая обработка данных проводилась с использованием программы Microsoft Office Excel 2016 (Microsoft, США) и статистического пакета Statistica 12 (StatSoft inc., США). Количественные признаки описывались как медиана (Me) и квартили [ Q1; Q3], соответствующие 25 и 75 перцентилям.

## Этическая экспертиза

Исследование одобрено локальным этическим комитетом ЭНЦ №16 от 13.09.2023 г.

## РЕЗУЛЬТАТЫ

За десятилетний период в детском хирургическом отделении было прооперировано 980 детей с узловыми образованиями щитовидной железы. Примечательно, что по результатам патоморфологического исследования более половины удаленных образований (51,6%; n=506) оказались злокачественными. В остальных случаях была диагностирована фолликулярная аденома (n=234) и фолликулярно-узловая болезнь ЩЖ (n=240).

Особый интерес представляет структура злокачественных опухолей. Наиболее распространенной была дифференцированная карцинома щитовидной железы, диагностированная у 472 из 506 детей со злокачественными новообразованиями ЩЖ. Подавляющее большинство детей имели папиллярную карциному — 88,5% (n=448). Фолликулярная карцинома установлена в 4,8% (n=24) случаев (рис. 3). Медиана возраста на момент постановки диагноза ДКЩЖ составила 15 лет [ 13; 16], при этом соотношение девочек и мальчиков — 2,5:1. Медиана длительности динамического наблюдения для детей с ДКЩЖ составила 12 месяцев [ 1,0; 36,0];

**Figure:**
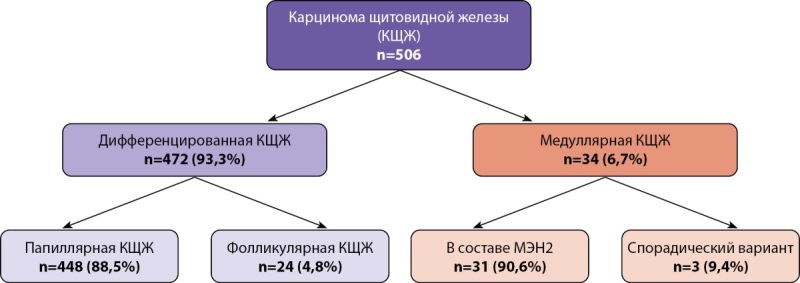
Рисунок 3. Структура злокачественных новообразований щитовидной железы у детей по данным ЭНЦ за 2015–2024 гг.

Медуллярная карцинома встречалась значительно реже ДКЩЖ — 34 случая (6,7%). При этом в подавляющем большинстве (n=31) была составляющей синдромов множественной эндокринной неоплазии (МЭН2А или МЭН2В), что подчеркивает особое значение генетического консультирования и молекулярной диагностики узлового зоба у детей.

Дооперационная диагностика. Всем пациентам с подтвержденной ДКЩЖ, по данным патоморфологического заключения (n=472), до оперативного лечения проводилась тонкоигольная аспирационная биопсия под контролем УЗИ с цитологической оценкой по системе Bethesda. Уже на этом этапе злокачественный характер образования у значительной части пациентов не вызывал сомнений: в 29% случаев (n=137) выявлена категория Bethesda V, в 28% (n=132) — Bethesda VI. Интересно, что почти треть детей с подтвержденной впоследствии ДКЩЖ имела цитологический результат, трактуемый как Bethesda IV — 30,9% (n=146).

Отдельно хочется отметить пациентов, оперированных не по поводу подозрительных в отношении злокачественности узловых образований, а в связи с наличием клинически значимых показаний к операции (размер узла более 3 см, компрессия трахеи, токсическая аденома). Дифференцированная карцинома в этой группе пациентов выявлена в категориях Bethesda III — 3,6% (n=17), Bethesda II — 8,3% (n = 39) и Bethesda I — 0,2% (n=1). Однако указанные показатели не отражают истинную частоту злокачественности в данных цитологических категориях, поскольку рассчитаны исключительно среди пациентов, подвергнутых оперативному лечению, а не среди всей совокупности узловых образований.

Метастатическое поражение регионарных ЛУ шеи до операции выявлено у 99 пациентов (21%). Во всех случаях наличие метастазов в ЛУ было подтверждено цитологическим исследованием (проводилась ТАБ со смывом на тиреоглобулин).

Хирургическая тактика при ДКЩЖ. Объем оперативного вмешательства определялся с учетом цитологических данных, ультразвуковой картины, размеров узла, степени регионарного распространения, а также наличия герминальных мутаций, ассоциированных с узлообразованием и риском развития ДКЩЖ.

Гемитиреоидэктомия с ипсилатеральной лимфаденэктомией выполнена 211 пациентам; в 30% случаев (n=63) по данным патоморфологического исследования потребовалась окончательная тиреоидэктомия.

Тиреоидэктомия с центральной лимфаденэктомией проведена 162 пациентам.

Тиреоидэктомия с центральной и боковой лимфаденэктомией выполнена 99 детям.

## Послеоперационное лечение и исходы при ДКЩЖ

Всем детям проведена стратификация риска рецидива и прогрессирования по критериям ATA [21]. Радиойодтерапия выполнена 229 детям (48,5%), у 13 из них при проведении посттерапевтической сцинтиграфии выявлены отдаленные метастазы в легкие.

Рецидив карциномы щитовидной железы зафиксирован в 5,1% случаев (n=24), вне зависимости от объема операции и проведенной радиойодтерапии.

Рецидивы после оперативного лечения: у 2 пациентов через 16 и 21 месяц после проведения гемитиреоидэктомии были обнаружены рецидивы опухоли в лимфоузлах боковой клетчатки шеи на стороне поражения. В обоих случаях потребовалось проведение окончательной тиреоидэктомии с центральной и боковой лимфаденэктомией. У 4 детей после тиреоидэктомии рецидив опухолевого роста обнаружен чрез 8, 10, 11 и 31 месяц соответственно. Причем у 3 пациентов метастазы были в боковых лимфоузлах шеи, у 1 — в лимфоузле центральной клетчатки. После повторного хирургического вмешательства все дети получили терапию радиоактивным йодом.

Рецидивы после радиойодтерапии: у 3 пациентов медиана возникновения рецидива после РЙТ составила 26 месяцев [ 22; 27], детям было проведено повторное лечение радиоактивным йодом. У 15 пациентов, получивших ранее РЙТ, медиана возникновения метастазов в боковые ЛУ шеи составила 8 месяцев [ 6; 21]. Учитывая диаметр измененных ЛУ, этой группе детей первоначально потребовалось проведение повторного оперативного лечения в объеме боковой лимфаденэктомии.

Следует заметить, что у 78 из 472 детей (16,5%) с дифференцированной карциномой щитовидной железы заболевание проявлялось более тяжелым клиническим течением. Для этих детей характерным являлось экстратиреоидное распространение опухоли, множественное метастатическое поражение лимфатических узлов шеи, проведение повторных хирургических вмешательств, наличие отдаленных метастазов и/или необходимость повторных курсов радиойодтерапии (рис. 4). Особый интерес представляют четыре случая радиойодрезистентности. В одном из них стабилизации процесса удалось достичь посредством применения таргетной терапии ленватинибом: за 4 года наблюдения прогрессирования не зарегистрировано, при этом таргетная терапия отличалась высокой эффективностью и удовлетворительной переносимостью. У других трех пациентов наблюдалась самостоятельная стабилизация заболевания.

**Figure:**
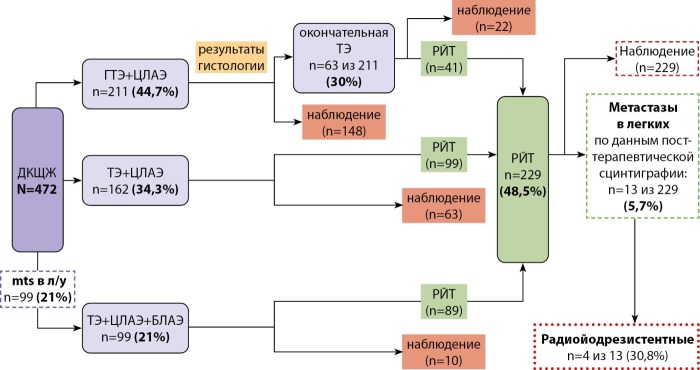
Рисунок 4. Данные хирургического и комбинированного лечения детей с дифференцированной карциномой щитовидной железы в ЭНЦ за 2015–2024 гг.

## Молекулярно-генетическое исследование

С 2018 по 2024 гг. молекулярно-генетическое тестирование, направленное на поиск герминальных мутаций, было проведено 66 детям с ДКЩЖ при наличии отягощенного семейного анамнеза и/или клинических признаков наследственных синдромов, ассоциированных с повышенным риском развития ДКЩЖ. Патогенные варианты обнаружены в 53,1% (n=34) случаев. Спектр мутаций оказался гетерогенным, что отражает сложность молекулярных механизмов канцерогенеза ЩЖ в детском возрасте. Наиболее часто встречались изменения в генах DICER1 (n=12), PTEN (n=4) и APC (n=4). Реже выявлялись мутации в TG (n=2), DUOX2 (n=2), AKAP9 (n=1), BBS7 (n=1), BRCA1 (n=1), BRCA2 (n=1), NF1 (n=1), TPO (n=1), AIP (n=1), PRKCA (n=1), CDKN2A (n=1), CHEK2 (n=1) (рис. 5).

**Figure:**
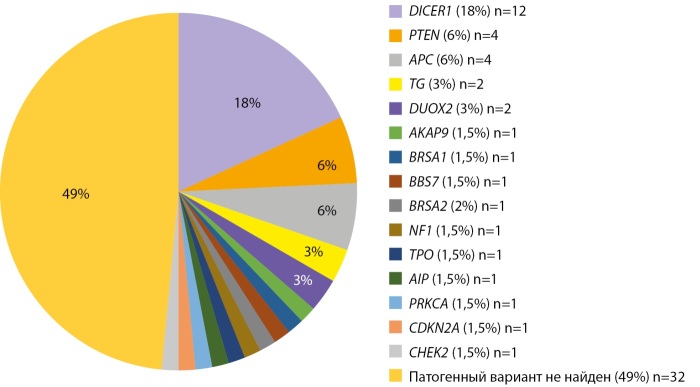
Рисунок 5. Результаты молекулярно-генетического исследования 66 детей с дифференцированной карциномой щитовидной железы при наличии отягощенного семейного анамнеза и/или клинических признаков наследственных синдромов, ассоциированных с повышенным риском развития ДКЩЖ.

У четырех пациентов с длительной биохимической и/или структурной персистенцией заболевания выявлены различные герминальные мутации: DICER1 (c.3251C>A), APC (c.4447C>A), PRKCA (c.1405G>A), CHEK2 (c.592+3A>T).

## ОБСУЖДЕНИЕ

Представленные результаты 10-летней клинической практики в стенах одного медицинского центра демонстрируют высокую долю злокачественных опухолей (51,6%) у детей и подростков, оперированных по поводу узловых образований ЩЖ. Высокая частота выявления карциномы щитовидной железы обусловлена, с одной стороны, спецификой когорты: в нее включены только пациенты с узловыми образованиями, перенесшие хирургическое лечение, с другой — концентрацией наиболее сложных клинических случаев в специализированном центре. Тем не менее полученные данные подчеркивают необходимость особого внимания к узловым образованиям ЩЖ у детей: обнаружение узла у ребенка должно расцениваться как фактор высокого онкориска и требовать более активной диагностической тактики по сравнению со взрослыми пациентами [1][8][22].

Несмотря на 100-процентную общую выживаемость детей с ДКЩЖ, значительная часть пациентов имеет длительную историю болезни, включающую повторные хирургические вмешательства, проведение одного или нескольких курсов радиойодтерапии и развитие сопутствующих осложнений. В нашей когорте уже на дооперационном этапе у 21% пациентов выявлено метастатическое поражение лимфоузлов боковой клетчатки шеи, 48,5% детей получили терапию радиоактивным йодом, у 5,1% зафиксированы рецидивы, а у 16,5% пациентов заболевание сохранялось после первичного лечения в виде биохимической и/или структурной персистенции. Все это отражает характерные особенности ДКЩЖ у детей, когда заболевание часто манифестирует в более распространенной форме по сравнению со взрослыми, при этом прогноз по выживаемости остается благоприятным даже у детей с отдаленными метастазами, которые нередко сохраняются на протяжении многих лет [14][15][23]. Данные результаты подчеркивают необходимость централизованного, мультидисциплинарного ведения детей с ДКЩЖ [1][10]. Вопросы оптимального объема хирургического вмешательства, минимизации связанных с ним осложнений и критериев назначения РЙТ остаются наиболее дискуссионными, особенно в отношении пациентов с промежуточным риском рецидива карциномы щитовидной железы.

Значительная частота регионарного метастазирования подтверждает оправданность расширенных хирургических вмешательств у детей с подозрением на лимфогенную диссеминацию. Эти данные согласуются с результатами международных исследований, подчеркивающих выраженную склонность детской ДКЩЖ к метастазированию в лимфатические узлы, что диктует необходимость углубленной предоперационной оценки для точного выбора объема операции [1][8][10].

Отдельного внимания заслуживают пациенты с отдаленными метастазами в легкие. У 4 из 13 детей (30,8%) отмечена радиойодрезистентность. В одном случае применение ленватиниба позволило достичь стойкого контроля заболевания на протяжении четырех лет наблюдения при удовлетворительной переносимости терапии. Подобные результаты демонстрируют перспективность применения таргетной терапии в педиатрической онкологии щитовидной железы и подчеркивают необходимость дальнейших клинических исследований в этом направлении [24][25].

Молекулярно-генетический анализ показал, что у детей с дифференцированной карциномой щитовидной железы наиболее часто выявлялись герминальные мутации в генах DICER1, PTEN и APC. Известно, что мутации DICER1 ассоциированы с широким спектром опухолей, включая узловые образования ЩЖ разной степени злокачественности [26][27], а изменения в PTEN рассматриваются как фактор риска развития ДКЩЖ в детском возрасте [28]. Полученные результаты подчеркивают необходимость включения молекулярного тестирования в предоперационный или ранний послеоперационный этап обследования, особенно при подозрении на наследственные синдромы, ассоциированные с развитием ДКЩЖ. Использование молекулярных панелей позволяет повысить точность стратификации риска и оптимизировать объем оперативного вмешательства [29].

Таким образом, наш опыт подтверждает ключевые положения современных европейских (ETA-2022) и российских (2024) рекомендаций: целесообразность органосохраняющего подхода, выборочного применения РЙТ, внедрение молекулярно-генетического анализа и ведение пациентов в специализированных центрах [1][8].

Ограничения исследования. Необходимо признать, что наша работа является ретроспективным одноцентровым анализом пациентов, оперированных в профильном национальном центре; отраженные здесь показатели (включая долю злокачественности и частоту выявленных мутаций) подвержены реферальному и селекционному смещению и потому не могут быть напрямую экстраполированы на популяцию в целом. Кроме того, молекулярное тестирование, направленное на поиск герминальных мутаций, ассоциированных с развитием ДКЩЖ, выполнено лишь у части пациентов (n=66), что ограничивает выводы о распространенности генетических событий в общей когорте. Наконец, в ретроспективном дизайне возможны пропуски данных и неоднородность протоколов ведения в разные годы (2015–2024).

Сильные стороны. Большой объем клинического материала, единообразие диагностических и хирургических протоколов в одном центре, а также наличие детальной морфологической и молекулярной информации для значительной подвыборки делают работу весомым вкладом в понимание клинической характеристики узловой патологии щитовидной железы у детей и подростков.

## Перспективы и рекомендации для дальнейших исследований

Наш опыт подтверждает необходимость централизованного и многопрофильного подхода к детям с узловой патологией ЩЖ: ранняя высококачественная УЗ-диагностика, стандартизированная цитологическая оценка, расширенное молекулярно-генетическое тестирование и обсуждение на мультидисплинарных консилиумах помогают корректно определить объем операции и последующую тактику (включая показания к РЙТ и объем исследований и частоту наблюдений).

## ЗАКЛЮЧЕНИЕ

Дифференцированная карцинома щитовидной железы у детей имеет свои особенности клинического течения, что требует тщательной диагностики и мультидисциплинарного подхода. Высокий риск злокачественности, частота регионарного и лёгочного метастазирования вместе с характерными для детских опухолей генетическими особенностями, аргументируют необходимость активного использования комбинированной предоперационной оценки (УЗИ, ТАБ, молекулярное тестирование) и персонализированного выбора объёма хирургического вмешательства у педиатрических пациентов в профильных центрах.

## ДОПОЛНИТЕЛЬНАЯ ИНФОРМАЦИЯ

Источники финансирования. Работа выполнена в рамках государственного задания Минздрава РФ НИОКТР №123021000039-3.

Конфликт интересов. Авторы декларируют отсутствие явных и потенциальных конфликтов интересов, связанных с содержанием настоящей статьи.

Участие авторов. Все авторы одобрили финальную версию статьи перед публикацией, выразили согласие нести ответственность за все аспекты работы, подразумевающую надлежащее изучение и решение вопросов, связанных с точностью или добросовестностью любой части работы.
